# The Impact of Transformational Leadership on Nurses’ Organizational Commitment: A Multiple Mediation Model

**DOI:** 10.3390/ejihpe10010021

**Published:** 2019-12-09

**Authors:** Kamran Iqbal, Tehreem Fatima, Muhammad Naveed

**Affiliations:** 1 Department of Management Sciences, University of Lahore, Sargodha 40100, Pakistan; tehreemfatima1010@gmail.com (T.F.); qm.naveed@outlook.com (M.N.); 2 Department of Management Sciences, Bahria university Islamabad, Islamabad 44000, Pakistan

**Keywords:** transformational leadership, psychological empowerment, psychological well-being, organizational commitment, nursing sector

## Abstract

This study proposed that the transformational style of nursing staff supervisors inculcate commitment amongst nurses. Moreover, psychological empowerment and psychological well-being were posited as multiple meditators in the above-mentioned association, based on the tenant of conservation of resource (COR) theory. The authors have collected the survey data from the sample of (n = 299) hospital nurses working in Sargodha district of Pakistan. The bootstrap results have supported the direct, as well as indirect relationships. These findings imply that when the nursing staff perceives their leader as transformational, their psychological empowerment is enhanced, and they have higher well-being, which in turn increases their commitment to their hospitals. This study offers a better understanding of psychological states that mediate transformational leadership and organizational commitment linkage.

## 1. Introduction

Nursing is considered as the central component of the healthcare sector in all parts of the world. However, in a developing country like Pakistan, it has a significant contribution to health care. Pakistan’s estimated population is over 200 million, making it the fifth most populated nation in the world. More than half of the people do not have access to essential health and educational facilities. The availability of nurses is insufficient to meet the requirement of healthcare. Although nurse to population ratio improved from 1:32,000 in 1960 to 1:5199 by 1997 [1], it is still very low, compared to developed nations. Improvement in quality of patient care is a primary goal of health care organization [2]. Nurses’ commitment to their hospitals is critical for the maintenance of high-quality safety and health care environment [3]. Because nurses work closely with patients in hospitals, their commitment to their hospital is highly crucial for quality assurance. The term “organizational commitment” refers to the attachment between employee and organization [4]. According to [5], committed employees put more effort into achieving organizational goals and objectives. Also, organizational commitment has been associated with several employee attitudes and behaviors, for instance, job performance [6], job satisfaction [7] and job turnover [8].

Organizational commitment is believed to be an outcome of the positive exchange relationship between an organization and its employees [9]. Research shows that increase in salary and monetary benefits are not appropriate methods to resolve the issue of nurses’ shortage in hospitals; instead, organizations should focus on non-monetary factors to motivate nursing staff [10]. Amongst these non-monetary factors, leadership is found to be an antecedent of employee commitment. Transformational leadership has surfaced as one of the most important leadership styles that foster higher levels of commitment in employees [11]. It is defined as “a process through which leaders and followers raise one another to higher levels of morality and motivation” [12] (p. 20). According to Bass [13], transformational leadership style leads to a higher level of motivation and performance in employees than transactional leadership style. Shamir et al. [14] pointed out that these kinds of leaders convey salient messages to their employees about the impact and importance of the group’s task and how their efforts contribute toward a group success. A transformational leader is one who “looks for the potential motives in followers, seeks to satisfy higher needs, and engages the full person of the follower” [12] (p. 4). Such leaders lead their employees through motivation, inspiration, and inculcate valuable and positive changes in their workers. They inspire individuals to perform at an unexpected level and produce exceptional outcomes. By doing so employee’s attitudes are changed in ways that enhance their motivation to achieve organizational goals. Prior studies on transformational leadership have linked it with employee well-being [15], project success [16], job satisfaction [17], patient safety [17], job crafting [18,19], employee creativity [20], knowledge sharing [21], performance outcomes [22], managerial performance [23], work engagement [24], proactive work behavior [24], organizational performance [25], and organizational citizenship behavior [26]. In nursing, this leadership concept has been linked with higher team performance and better patient care [27]. Recently, transformational leadership style is found to be effective in leading nursing staff and has proven to be a precursor of positive organizational outcomes. It is suggested as an appropriate leadership style, to overcome the issue of nurse retention [17]. 

Even though there is a well-established empirical and conceptual association of transformational leadership with organizational commitment [11], researchers are still in quest of clarifying the mechanisms that link these two variables. In this study, we aim to depart from the use of single mediators of, i.e., psychological empowerment [11] and leader-member exchange [28], and introduce multiple explanatory paths linking transformational leadership to organizational commitment. One is through psychological empowerment and the other is through the mechanism of psychological well-being. For this purpose, we build on the CORperspective and argue that the transformational leaders are able to inculcate higher psychological empowerment in the followers by escalating the level of meaningfulness they attribute to their work, having necessary capabilities to perform the assigned tasks, and having a sense of influence over one’s environment [11]. Moreover, they are also capable of bringing a sense of higher psychological well-being in employees through support and motivation, which enhances the satisfaction and feeling of accomplishment in the followers [29]. These positive psychological states, in turn, create a stronger bond and attachment to organization, increasing the levels of organizational commitment.

## 2. Theoretical Background and Hypotheses Development

The tenant of the COR theory states that employees need resources for effective work performance and retention. These resources may be physical, social, or psychological, derived from the factors present in their work contexts [30]. Leaders are an important source of imparting resources to their followers; a good quality association between leaders and followers results in more resourceful employees [31]. Building on the theoretical conception of COR, it is argued that the inspiration, motivation, support and individualized consideration given by the transformational leader makes their followers more resourceful in terms of empowerment [32] and well-being [33] which ultimately leads to positive organizational outcomes, i.e., organizational commitment. Employees that feel psychologically independent and attribute more meaning to their work have a higher level of involvement in organizational activities [34]. Similarly, the psychological well-being at work is yet another important resource that employee’s value, and it enhances their capacity to be retained and have a high attachment to the organization [35].

**Figure 1:**
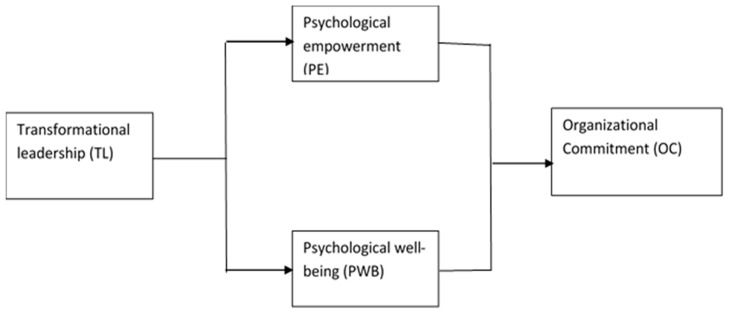
The hypothesized mediation model.

Our proposed model focuses on the examination of the nexus between transformational leadership, psychological empowerment, and organizational commitment, described in Figure 1. This study helps to understand the black box between transformational leadership and organizational commitment in the nursing sector. The proposed model suggests that transformational leadership stimulates psychological empowerment and psychological well-being, which in turn influence nurses’ organizational commitment. 

### 2.1. Transformational Leadership and Organizational Commitment

Organizational commitment is defined as “the relative strength of an individual’s identification with and involvement in a particular organization” [4] (p. 27). Organizational commitment (OC) shows the degree of association that employees have with their organization [36]. Research has uncovered that organizational commitment leads to many benefits for both employees and the organization [37]. Rowden [38] found that higher levels of commitment lead to job security, career advancement, and increased rewards for the employees. Prior studies proclaim the association between transformational leadership and organizational commitment by using various contextual settings [39,40,41,42] and obtain divergent findings [43,44]. Bushra et al. [45] advocate that transformational leadership is a potential determinant of organizational commitment, having studied banking sector employees in Pakistan. Transformational leaders induce the voice of their followers in the policymaking process and help them to increase their potential [46]. When managers consider the needs of their followers and appreciate their efforts to apply new approaches to solve work-related problems, it motivates them to get more engaged in their job, and this results in the higher commitment of staff to their organization [44]. Based on the substantive review, the following hypothesis has been deduced:

**Hypothesis** **1** **(H1).** *There is a positive relationship between transformational leadership and organizational commitment.*

### 2.2. Transformational Leadership and Psychological Empowerment

The concept of employee empowerment has been extensively studied during the last two decades in different settings and contexts, such as information technology organizations and academics [47], social workers [48], banking managers [49], public welfare caseworkers [50], manufacturing firms [51], hotel staff [52] and nurses [11]. Spreitzer [53] defined psychological empowerment as “increased intrinsic task motivation manifested in a set of four cognitions reflecting an individual’s orientation to his or her work role: competence, impact, meaning, and self-determination” (p. 1443). Organizations use empowerment as an import tool by delegating the responsibility and freedom to their employees so that they use their creative ideas to add value in products and services offered by these organizations [54]. Psychological empowerment is one of the main factors that distinguishes the transformational leadership style from other leadership styles [55]. Therefore, it can be proposed that the transformational leadership style is a possible predictor of psychological empowerment. Past studies proclaim a significantly positive nexus between transformational leadership and psychological empowerment [47,56,57]. Therefore, we hypothesize that:

**Hypothesis** **2** **(H2).** *Transformational leadership is positively related to psychological empowerment*.

### 2.3. Psychological Empowerment and Organizational Commitment

Empowered employees consider themselves to be more competent and influential in their organizations and at their job. It is posited that when employees perceived that their organization is empowering them, they are more likely to respond with a higher level of organizational commitment [58]. Empowered employees perceive themselves to have an impact on their organizations and in their jobs [11]. In this way, empowerment motivates employees to perform at the highest level, and exhibit a higher organizational commitment to achieving organizational goals [59]. We, therefore, hypothesize:

**Hypothesis** **3** **(H3).** *Psychological empowerment is positively related to organizational commitment.*

Based on the literature, it is evident that the transformational leadership style fosters a higher level of psychological empowerment in employees that thrive from organizational commitment. So, it is hypothesized that:

**Hypothesis** **4** **(H4).** *Psychological empowerment mediates the link between transformational leadership and organizational commitment*.

### 2.4. Transformational Leadership and Psychological Well-Being

Psychological well-being is an essential dimension of the overall well-being of employees [60]. Psychological well-being is the state of happiness and satisfaction with experiences at work and life in general, utility, sense of achievement, and belongingness. Psychological well-being has been studied with several job-related variables, i.e., with the working environment [61,62], job insecurity [63], job burnout [64], employee performance [65], and psychological capital [66]. Transformational leaders can help employees to reduce stress levels through their mentoring function [67]. Bass and Riggio [68] highlighted that transformational leaders help their subordinates to achieve challenging goals through their continuous support and motivation. McVicar [33] suggested that transformational leadership influences well-being of employees, especially in the health sector, where the environment is highly stressful. We expect a definite link between transformational leadership and psychological well-being. Hence, we hypothesize that:

**Hypothesis** **5** **(H5).** *Transformational leadership has a positive impact on psychological well-being.*

### 2.5. Psychological Well-Being and Organizational Commitment

Nielsen et al. [69] argue that transformational leaders enhance self-efficacy in employees and increase trust in management, which results in higher psychological well-being. Also, researchers have proclaimed a substantial positive link among well-being and employee outcomes, such as organizational commitment [70]. Hence, we expect a positive relationship between psychological well-being and organizational commitment. As a result, we hypothesize:

**Hypothesis** **6** **(H6).** *Psychological well-being has a positive impact on organizational commitment.*

We propose that transformational leadership is linked with psychological well-being and that psychological well-being is positively related to organizational commitment. Thus, we also propose a mediating hypothesis:

**Hypothesis** **7** **(H7).** *Psychological well-being mediates the link between transformational leadership and organizational commitment.*

## 3. Methodology

### 3.1. Sample and Procedure

We focused on the nursing staff of private and public hospitals of Sargodha District, the 12th largest city in Pakistan with a population of over two million. It consists of more than 30 big hospitals. The collection of the data was self-administered, and participants were assured about the confidentiality of their responses. Self-administration helps the researcher to make sure that participants are giving their responses after proper clarification and understanding of the contents. This study adopted the convenience sampling technique. A total of 618 questionnaires were circulated, 330 were returned. Amongst the returned questionnaires, a few questionnaires were partially filled and contained multiple responses, these were removed. Therefore, only 299 (48%) were used for the final analysis. The surveys were conducted in English as it is the medium of instruction in all higher education institutes in Pakistan, and English is well understood among the nursing staff of hospitals in Pakistan. A cover letter showing the purpose of the visit was attached in front of the questionnaire. Ethical approval was obtained from the district health authority. The respondents were told about the aims and objectives of the study. The participation of the respondents was voluntary, and they were assured data confidentiality Data were analyzed using 5000 bootstrapped samples and run though model 4 by using SPSS macro, which was developed by Preacher and Hayes [71] to test all hypotheses. All measures were evaluated at five-point Likert scale ranging from 1 = strongly disagree to 5 = strongly agree. The sample consisted of 52.5% males and 47.5% females. From the total respondents, 64% were married, and 36% were single. A total of 73% were aged less than 35, 30% less than 25 years old, 17% between ages 36 to 50, while only 10% were aged more than 50 years old.

### 3.2. Measures 

#### 3.2.1. Transformational Leadership

Transformational leadership was measured by using seven items taken from [72]. Sample items were “my supervisor treats staff as individuals, supports and encourages their development”; “my supervisor encourages thinking about problems in new ways and questions assumptions.” The Cronbach’s alpha value for this variable was 0.703.

#### 3.2.2. Organizational Commitment

The questionnaire of organizational commitment was adopted from Meyer et al. and Meyer and Allen [73,74]. The scale consisted of 5 items. Sample items were “I would be happy to work at my organization until I retire”; “I would be happy to work at my organization until I retire” The Cronbach’s alpha value for this variable was reported as 0.766.

#### 3.2.3. Psychological Empowerment

The psychological empowerment was measured by adopting a scale developed from Spreitzer [53]. The questionnaire contains 12 items, for example, “I can decide on my own how to go about doing my work”; “I have considerable opportunity for independence and freedom in how I do my job” The Cronbach’s alpha value for this variable was 0.863.

#### 3.2.4. Psychological Well-Being

The measure of psychological well-being was adapted from Diener et al. [75]. The measure consisted of 8 items, for instance, “I am engaged and interested in my daily activities”; “I actively contribute to the happiness and well-being of others.” The Cronbach’s alpha value for this variable was 0.792. 

## 4. Results

### 4.1. Descriptive Statistics and Correlation 

Table 1 exhibits descriptive statistics (which include mean, standard deviation, and correlations) of the demographical variables. Transformational leadership was found to be significantly correlated to psychological empowerment (r = 0.575, *p* < 0.01), psychological well-being (r = 0.621, *p* < 0.01) and organizational commitment (r = 0.529, *p* < 0.01). A significant correlation was found between and psychological empowerment and organizational commitment (r = 0.674, *p* < 0.01), and between psychological well-being and organizational commitment (r = 0.617, *p* < 0.01). 

### 4.2. Confirmatory Factor Analysis

Before testing the hypotheses, a series of confirmatory factor analyses were run to verify the best suitable measurement model [76]. The result of the proposed model showed excellent model fit, χ^2^ = 842.52, CFI = 0.947, TLI = 0.931, and RMSEA= 0.057, as compared to another possible model. Also, the factor loadings of all indicators loaded significantly, and their values were greater than 0.50. On the whole, the confirmatory factor analysis results revealed that the proposed model has adequate discriminant validity (Table 2). 

### 4.3. Mediation Analysis 

#### 4.3.1. Mediating Effect of Psychological Empowerment

Regression results have been used according to instructions given by Barron and Kenny and preacher and Hayes [71,77]. Table 3 shows a significant total effect between the transformational leader and organizational commitment (B = 0.2686, *p* < 0.001). Hence, the first condition of mediation is fulfilled, as per Barron and Kenny [71]. Next, there is a significant positive relationship between transformational leaders and psychological empowerment (B = 0.2764, *p* < 0.001). These results support our second hypothesis, H2, and the second condition of mediation. Next, a significant and positive effect of psychological empowerment and organizational commitment is observed (B = 0.5829, *p* < 0.001). Hence, our third hypothesis, H3, and the third condition of mediation are supported. Fourth, a significant positive link between transformational leadership and organizational commitment has been reduced substantially and has become B = 0.1075, *p* < 0.001. It is still significant and supports our first hypothesis, H1. Finally, mediation was tested, based on the significance of the indirect effect as directed by Preacher and Hayes [71]. In this study, the authors first applied four conditions of Barron and Kenny [77], and then we examined the significance of the indirect effect by bootstrapping the sampling distribution. The results revealed that indirect effect of transformational leadership on organizational commitment is also found to be significant (Sobel Z = 0.1611, *p* < 0.001). The bootstrap result on a 95% level of confidence for all confidence intervals did not contain zero (Lower levels of confidence interval (LLCI) = 0.1238, Upper levels of confidence interval (ULCI) = 0.2036). Hence these results have also supported our fourth hypothesis, H4. 

#### 4.3.2. Mediating Effect of Psychological Well-Being

Table 4 shows that the total effect of transformational leadership on organizational commitment is significant (B = 0.2686, *p* < 0.001). Next, there is a significant positive relationship between transformational leadership and psychological well-being (B = 0.2783, *p* < 0.001). Hence, the fifth hypothesis, H5, is accepted. Next, significant and positive effects of psychological well-being and organizational commitment were noticed (B = 0.5322, *p* < 0.001). This supports the sixth hypothesis H6, and the third condition of mediation are supported. Fourth, a significant positive link between transformational leadership and organizational commitment has been reduced substantially and has become (B = 0.1205, *p* < 0.001). So H7 is partially supported. The indirect effect of transformational leadership on organizational commitment was also found to be significant (Sobel Z = 0.1481, *p* < 0.001). As the bootstrap result on a 95% level of confidence for all confidence intervals did not contain zero (LLCI = 0.0993, ULCI = 0.1930). Hence these results also support our seventh hypothesis, H7.

## 5. Discussion

The present study observed the link of transformational leadership with organizational commitment through the underlying role of psychological empowerment and well-being. We aimed to extend the general positive effect of the transformational leadership style of nursing staff supervisors on the commitment of nurses working in the context of the targeted population. The results of our study generated important findings based on the COR theoretical lens. 

First of all, the direct association of transformational leadership and organizational commitment was supported, based on the results. Leadership, in general, is considered as an antecedent of organizational commitment. Specifically, transformational leadership is found to be a precursor of organizational commitment in broad organizational and cultural settings [11]. The study found a positive relationship between transformational leadership and organizational commitment, which is consistent with previous findings [11,78,79]. Transformational leaders can induce commitment in their followers by inspiring them, promoting and supporting innovation, giving individual consideration, and modeling the desired behaviors. As per the COR perspective, leaders provide resources to followers that are necessary to engage in positive organizational outcomes [31]. In this way, transformational leadership results in a higher level of intrinsic value that the followers then place on goal achievement, in turn, this fosters an overall commitment to the attainment of commonly held organizational visions [28].

Though the connotation that transformational leadership and organizational commitment have theoretical and empirical existence [11], researchers have increasingly called for the clarification of the underlying factors that link these two constructs [28]. We have extended the direct association in transformational leadership and organizational commitment through the role of psychological empowerment. The results support this hypothesis, which confirms the study of Balaji and Krishnan [80]. This is through the creation of higher psychological empowerment in the followers, by increasing the level of meaningfulness they attribute to their work, having the necessary capabilities to perform the assigned tasks, and having a sense of influence over their environment [11]. Moreover, psychologically empowered employees are found to be more committed [81]. Based on COR, transformational leaders provide their followers with the needed autonomy and meaningfulness at work. This constitutes an important psychological resource to induce the state of organizational commitment [11,31]. This kind of psychological empowerment persuades a higher level of employee involvement and attachment to their organizations [82]. 

Another way that transformational leaders promote commitment in their followers is by enhancing the level of psychological well-being. So, we proposed psychological well-being as an explanatory mechanism in clarifying the transformational leadership and organizational commitment relationship. The cohesive nature of transformational leadership and psychological well-being was supported by the results, as well as aligned with studies expounded in the literature [83]. The support and motivation imparted by the transformational leaders make the followers more satisfied, accomplished, and positive at their work [29]. This positive psychological state, in turn, creates a stronger bond and attachment to organization, increasing the levels of organizational commitment that is supported by the notion of COR [31]. Our results supported the results of [70]; concluding a significant association between psychological well-being and organizational commitment. Thus, the followers have higher psychological well-being when they are led by a transformational leader that improves their organizational commitment.

The main thrust of this investigation was to test whether psychological empowerment and psychological well-being were the routes through which transformational leadership determine the commitment of nursing staff. Theoretically, this study is unique in the sense that it has identified multiple mediating mechanisms linking transformational leadership to organizational commitment through psychological empowerment and psychological well-being. This has extended the field by moving beyond the use of a single mediator of psychological empowerment [11] and leader-member exchange [28]. We have contributed to the literature by using a COR perspective to explain transformational leadership relations with the follower outcomes. It is suggested that transformational leadership has an indirect influence on commitment through the psychological resources of empowerment and well-being [31]. Our results offer support to the notion that leaders’ actions have the capacity to shape employees’ behaviors and their work outcomes [84]. Thus, this study, based on the theoretical lens of COR, shows that transformational leaders are an essential source of imparting psychological resources to their followers [31]. It further emphasizes the importance of psychological empowerment and well-being as valuable resources that cause employees to have higher levels of organizational commitment [33,69]. A transformational leader acknowledges the needs of their followers and facilitates the provision of needed psychological resources to create a sense of empowerment and well-being in their followers [17]. These are much needed psychological resources for creating a commitment to the organization [85].

We offer practical implications for hospital management and nursing staff supervisors to practice transformational leadership style to foster a higher level of commitment amongst nurses. This study shows that transformational leadership style creates better outcomes in nurses as compared to overly-managed and inflexible authoritarian styles [86]. By using the transformational leadership style, nursing supervisors offer support, inspiration, individual-focused attention, and act as a role model for nurses, that fosters psychological empowerment and well-being. Nurses will have more independence and will be mentally satisfied while performing their job duties. In high-stress environments of hospitals, it is important that nurses have a higher level of empowerment and well-being to impart their services. This will enable the hospital management to improve staff retention [87] and create a sense of commitment among nurses that will not only reduce their turnover intention but also consolidate their attachment and involvement to their work and organization. Transformational leaders cultivate the feelings of empowerment and well-being in nursing staff that ultimately increase their organizational commitment. Hospitals have working environments that have a high prevalence of adverse circumstances, stress, and tension. Moreover, they face issues of nursing staff retention and shortage [17]. So, psychological empowerment and well-being are indispensable for boosting the organizational commitment of nursing staff that can be achieved through the practice of transformational style by nursing staff supervisors. 

## 6. Conclusions

The findings of this study suggest that transformational leadership, psychological well-being, and psychological empowerment affect nurses’ organizational commitment. Specifically, this study has determined the role of transformational leadership in nurses’ organizational commitment through two routes of psychological well-being and empowerment. This study concludes that both psychological mechanisms are significant, but the mechanism of psychological empowerment is slightly better than the mechanism of psychological well-being. We conclude that supervisors should have a good relationship with their subordinates to increase subordinates’ psychological empowerment and well-being. Hospital’s management should also work on leadership skills of staff members that are leading a team, so that they can positively impact their subordinate’s attitudes. We hope that future researchers will extend this study by examining the potential moderators that link transformational leadership to organizational commitment, i.e., occupational self-efficacy [88], social support [89], and power distance [90]. The more explanatory mechanism can be examined beyond psychological well-being and psychological empowerment, i.e., procedural justice [91], and the dimensions of organizational commitment could be examined as outcomes [28]. Since, data were collected from a single source and were collected at single time frame, there could be a problem of common method variance. Therefore, the use of longitudinal designs and data from more sources (i.e., supervisors) is suggested to further improve the rigor and authenticity of the findings in this study.

## Figures and Tables

**Table 1:** Correlations and descriptive statistics.

Variables	Mean	S. D	1	2	3	4	5	6	7
1. Gender	1.475	0.500	1						
2. Marital status	1.351	0.493	0.213 **	1					
3. Age	2.063	0.934	0.072	0.213 **	1				
4. Transformational leadership	4.164	1.541	−0.043	0.018	−0.068	1 (**0.703**)			
5. Psychological empowerment	2.571	0.741	0.014	0.064	0.011	0.575 **	1 (**0.863**)		
6. Psychological well-being	2.555	0.691	−0.009	−0.019	−0.007	0.621 **	0.696 **	1 (**0.792**)	
7. Organizational commitment	2.570	0.783	0.010	0.019	0.019	0.529 **	0.674 **	0.617 **	1 (**0.766**)

Notes: ** Significant level. *p* < 0.01, N = 299.

**Table 2:** Confirmatory factor analysis.

Model	χ^2^	CFI	TLI	RMSEA
Four-factor model (TL, PE, PWB, OC)	842.52	0.947	0.931	0.057
Three-factor model (combining PE and PWB into a factor)	1207.24	0.857	0.828	0.079
Two-factor model (combining PE, PWB, and OC into a factor)	1663.43	0.746	0.721	0.094
Two-factor model (combining TL and PE into a factor and, PWB and OC into a factor)	1684.22	0.727	0.701	0.112
Two-factor model (combining TL, PE and PWB)	1672.92	0.734	0.695	0.101
One factor model (combining all items into one factor)	1929.31	0.685	0.624	0.129

Notes: TL = Transformational leadership, PE = Psychological empowerment, PWB = Psychological well-being, OC = Organizational commitment.

**Table 3:** Testing of mediation (psychological empowerment).

Variable	B	SE	t	P	LLCI	ULCI
Transformational leadership regressed on organizational commitment: Total effect	0.2686	0.0250	10.7420	0.0000	0.2194	0.3178
Transformational leadership regressed on psychological empowerment	0.2764	0.0228	12.1056	0.0000	0.2315	0.3213
Psychological empowerment regressed on organizational commitment	0.5829	0.0539	10.8139	0.0000	0.4768	0.6889
Transformational leadership regressed on organizational commitment for Psychological empowerment: Direct effect	0.1075	0.0259	4.1490	0.0000	0.0565	0.1585
**The indirect effect of transformational leadership on organizational commitment through psychological empowerment**						
**Value**	**Boot SE**		**Boot LLCI**		**Boot ULCI**	
0.1611	0.0203		0.1238		0.2036	

**Table 4:** Testing of mediation (psychological well-being).

Variable	B	SE	t	P	LLCI	ULCI
Transformational leadership regressed on organizational commitment: Total effect	0.2686	0.0250	10.7420	0.0000	0.2390	0.3375
Transformational leadership regressed on psychological well-being	0.2783	0.0204	13.6440	0.0000	0.2383	0.3185
Psychological well-being regressed on organizational commitment	0.5322	0.0642	8.2915	0.0000	0.4059	0.6585
Transformational leadership regressed on organizational commitment for psychological well-being: Direct effect	0.1205	0.0288	4.1866	0.0000	0.0639	0.1771
**The indirect effect of transformational leadership on organizational commitment through psychological well-being**						
**Value**	**Boot SE**		**Boot LLCI**		**Boot ULCI**	
0.1481	0.0245		0.0993		0.1930	
